# Evaluating cortical excitatory and inhibitory activity through interictal intracranial electroencephalography in mesial temporal lobe epilepsy

**DOI:** 10.3389/fnins.2024.1424401

**Published:** 2024-09-24

**Authors:** Keisuke Nagata, Naoto Kunii, Shigeta Fujitani, Seijiro Shimada, Nobuhito Saito

**Affiliations:** ^1^Department of Neurosurgery, The University of Tokyo, Bunkyo-ku, Tokyo, Japan; ^2^Department of Neurosurgery, Jichi Medical University, Shimotsuke, Tochigi, Japan

**Keywords:** excitatory and inhibitory balance, electrocorticogram, gamma oscillation regularity, multiscale entropy, cortico-cortical evoked potential, epileptogenic zone

## Abstract

Gamma oscillation regularity (GOR) indicates the synchronization of inhibitory interneurons, while the reactivity of cortico-cortical evoked potentials (CCEPs) is supposed to reflect local cortical excitability. Under the assumption that the early response of CCEP near the stimulation site also indicates excitatory activity primarily mediated by pyramidal cells, we aimed to visualize the cortical inhibitory and excitatory activities using GOR and CCEP in combination and to use them to predict the epileptogenic zone (EZ) in mesial temporal lobe epilepsy (MTLE). In five patients who underwent intracranial electrode implantation, GOR and CCEP reactivity in the vicinity of the stimulation site was quantified. The interictal GOR was calculated using multiscale entropy (MSE), the decrease of which was related to the enhanced GOR. These parameters were compared on an electrode-and-electrode basis, and spatially visualized on the brain surface. As a result, elevated GOR and CCEP reactivities, indicative of enhanced inhibitory and excitatory activities, were observed in the epileptogenic regions. Elevated CCEP reactivity was found to be localized to a restricted area centered on the seizure onset region, whereas GOR elevation was observed in a broader region surrounding it. Although these parameters independently predicted the EZ with high specificity, we combined the two to introduce a novel parameter, the excitatory and inhibitory (EI) index. The EI index predicted EZ with increased specificity compared with GOR or CCEP reactivity alone. Our results demonstrate that GOR and CCEP reactivity provided a quantitative visualization of the distribution of cortical inhibitory and excitatory activities and highlighted the relationship between the two parameters. The combination of GOR and CCEP reactivities are expected to serve as biomarkers for localizing the epileptogenic zone in MTLE from interictal intracranial electroencephalograms.

## 1 Introduction

Disruption of the excitatory and inhibitory (EI) balance in the brain has attracted significant attention as a basic mechanism underlying epileptic seizures (Engel, 1996; Scharfman, 2007). In the simplest model, inhibitory interneurons control the excitatory firing of pyramidal cells through neurotransmitters such as glutamate and gamma-aminobutyric acid (GABA) (Bradford, 1995; Ziburkus et al., 2006). This interaction exhibits temporal and spatial dynamics within the brain, with networks involving both glutamate and GABA continuously rewiring over time (Mann and Paulsen, 2007). Although both in vivo and in vitro investigations of the excitatory and inhibitory activities of the neural cortex are available, studies that quantitatively evaluate these properties using human electrocorticograms (ECoGs) are limited. In this study, we focused on two parameters that could be calculated from interictal ECoG signals to evaluate the inhibitory and excitatory activities of neural circuits in patients with epilepsy: gamma oscillation regularity (GOR) and cortico-cortical evoked potential (CCEP) reactivity in the vicinity of the stimulation site.

GABAergic interneurons play a key role in maintaining the inhibitory activity in the cerebral cortex (Engel, 1995; Treiman, 2001). Interconnected networks formed by fast-spiking inhibitory interneurons exhibit rapid spiking activity following pyramidal cell firing, which can be recorded extracellularly at gamma oscillations ranging from 30 to 80 Hz (Buzsáki et al., 1983; Cardin et al., 2009; Tukker et al., 2007). Increases in GABAergic activity are closely related to epileptogenicity linked to interictal discharges following interneuron firing (Huberfeld et al., 2011), as well as epileptic gamma oscillations which could be suppressed by GABA blockers (Köhling et al., 2000). These findings have been confirmed in the human brain, and efforts have been made to utilize gamma oscillations to identify epileptogenic regions. A human ECoG study demonstrated that increased gamma oscillations occurred prior to epileptic spikes during interictal periods. Interictal gamma oscillations have high specificity for identifying seizure onset zones, although they are not sufficiently sensitive for precise focus localization (Ren et al., 2015).

When evaluating interneuronal gamma oscillations, it is not sufficient to simply calculate band power. Broadband high-gamma activity and low-gamma oscillations need to be considered separately, as the former could be physiologically involved in neurocognitive processes, while the latter are related to GABAergic inhibition (Buzsáki and Wang, 2012). Memory tasks increase physiological gamma activity while decreasing interictal spikes, indicating a trade-off between physiological and pathological gamma activity (Matsumoto et al., 2013).

To extract pathological gamma oscillations related to epileptogenicity, we introduced gamma oscillation regularity as an indicator to determine whether homologous waveforms were repeated in the gamma frequency band (Sato et al., 2021; Sato et al., 2017). To quantify GOR, an analytical tool termed multiscale entropy (MSE) was utilized (Costa et al., 2002). MSE measures the complexity of time-series data, and can be applied to biological waveforms, such as electrocardiograms and electroencephalograms (EEGs). MSE can quantify waveform complexities at each time scale (i.e., at each frequency band in EEGs and ECoGs) using windows of different time scales. A decrease in MSE indicates low complexity in the targeted frequency band of the time-series data, indicating that the waveform is composed of a repetition of the same information. This analytical method has previously been utilized in the study of Alzheimer’s disease (Ando et al., 2021), as well as other psychiatric disorders (Takahashi et al., 2009).

In patients with epilepsy, increased synchronization of inhibitory interneurons, manifesting as an enhanced GOR, was observed in epileptic foci during the interictal period. This pathological rhythm is detected in ECoG as a decrease in the MSE of the gamma band (Sato et al., 2017). The GOR, quantified by MSE, may be a relevant indicator of cortical inhibitory activity and a potential biomarker for the epileptogenic zone (EZ). During the interictal period, the GOR is most elevated in the seizure onset zone (SOZ) and moderately elevated in the surrounding EZ of the epileptic brain (Sato et al., 2017). Regions exhibiting increased GOR correspond to areas with interictal epileptic discharges and reduced accumulation on 123I-iomazenil single-photon emission computed tomography (Sato et al., 2022). GOR-guided resection has also been reported to improve seizure outcomes (Sato et al., 2021).

Meanwhile, cortical excitability, represented by CCEP in the vicinity of the stimulation site, has also been reported. The CCEP is an induced potential that occurs in a cortical region distant from the area where a single cortical stimulation is applied (Matsumoto et al., 2004). Its primary role is to estimate the functional connectivity of multiple brain regions by evaluating the reactivity in a cortical region adequately distant from the stimulation site. In previous connectivity studies, responses in regions adjacent to the stimulation site were recognized as stimulus artifacts and tended to be excluded from the analysis (Matsumoto et al., 2005). Conversely, other studies have reported that CCEP responses in the vicinity of the stimulation site reflect local cortical excitability in patients with epilepsy (Enatsu et al., 2012; Iwasaki et al., 2010; Kobayashi et al., 2017; Matsumoto et al., 2005). The CCEP waveform comprised an early negative peak (N1) and a late negative peak (N2). The amplitude of N1 in the cortical regions adjacent to the stimulation electrodes can vary depending on the excitability of the stimulation site. A higher amplitude results when stimulating the seizure onset zone than when stimulating regions not associated with ictal onset (Iwasaki et al., 2010; Matsumoto et al., 2005). This tendency was independent of epileptic foci location.

It should be noted that the cortical excitability obtained by electrical stimulation reflects not only excitatory, but also inhibitory activity. Kobayashi et al. demonstrated that high frequency activity overriding the N1 peak of CCEP is enhanced in SOZ while decreased in N2 (Kobayashi et al., 2017). This characteristic response to electrical stimulation is comparable to the excitatory synchronous neural firing followed by inhibitory activity observed in interictal epileptic discharges (Alarcón et al., 2012). We assumed that the early component of the CCEP recorded in the immediate vicinity of the stimulation site primarily reflected local cortical excitatory activity. This large and early potentials of the CCEP are primarily accounted for by volume-conducted potentials (VCP), which are generated at the stimulation site and attenuated with distance (Shimada et al., 2017). We focused on the power of the VCP and attempted to quantify it as a parameter of excitatory activity.

In this study, we hypothesized that epileptogenicity in patients with mesial temporal lobe epilepsy (MTLE) could be evaluated by increased GOR (i.e., decreased MSE) and increased CCEP reactivity in the vicinity of the stimulation site and even by their combination. We also aimed to spatially quantify the inhibitory and excitatory activities in the cerebral cortex during interictal periods based on the assumption that GOR and CCEP reactivity are indicators of cortical inhibition and excitation.

## 2 Materials and methods

### 2.1 Participants

The present study focused on patients with intractable epilepsy, aged 16 years and over, who underwent intracranial electrode implantation for diagnostic purposes. All participants underwent preoperative evaluations including head magnetic resonance imaging (MRI), nuclear imaging, and magnetoencephalography. Considering the neuroimaging examination results and long-term video-ECoG monitoring using intracranial electrodes, epileptic foci, surgical approaches, and resection boundaries were determined by a team of epileptologists during medical meeting discussions. From December 2018 to December 2020, 26 individuals underwent intracranial electrode implantation at the University of Tokyo Hospital. Of these, 12 were diagnosed with MTLE, of whom 11 underwent anterior temporal lobectomy, and hippocampectomy or multiple hippocampal resections. Participants who achieved seizure freedom for at least two years after surgery were included in the analysis, indicating that the seizure focus was undoubtedly included within the resection area. Before electrode implantation surgery, all participants completed the Wechsler Adult Intelligence Scale (WAIS), and those with a full-scale intelligence quotient (FIQ) less than 60 were excluded from further analyses to homogenize the neuropsychological background of the study population. Five participants who met our study criteria and completed two electrophysiological examinations during the electrode implantation period were finally included in this study. Table 1 presents the characteristics of the participants.

**TABLE 1:** Participant characteristics.

Participant no.	Age (years)	Implanted hemisphere	Number of total electrodes	Number of electrodes included in RA	FIQ	ASM	ASM during video ECoG
P1	50–59	Left	210	20	92	LEV 2000 mg LCM 300 mg ZNS 200 mg	ZNS 200 mg
P2	30–39	Left	210	20	105	LEV 3000 mg LCM 400 mg PER 4 mg	LCM 400 mg PER 4 mg
P3	50–59	Right	142	18	80	LEV 3000 mg LCM100 mg PER 6 mg CBZ 800 mg	PER 6 mg CBZ 800 mg
P4	16–19	Right	88	14	99	LEV 2000 mg LCM 200 mg PER 4 mg	LEV 2000 mg LCM 200 mg PER 4 mg
P5	50–59	Right	70	14	63	LCM 200 mg CBZ 600 mg CZP 1 mg	LCM 200 mg CBZ 600 mg CZP 1 mg

ASM, anti-seizure medication; CBZ, carbamazepine; CZP, clonazepam; ECoG, electrocorticography; FIQ, full-scale intelligence quotient; LCM, lacosamide; LEV, levetiracetam; PER, perampanel; RA, resection area; ZNS, zonisamide.

### 2.2 Electrode locations

**FIGURE 1:**
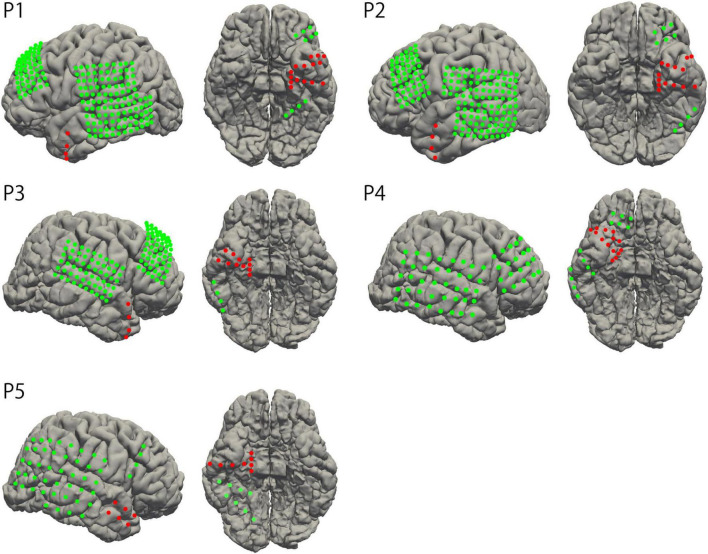
Electrode location. The locations of implanted electrodes were overlayed on standardized individual brains. The electrodes in the region of surgical intervention are represented as red dots.

Grid- and strip-type subdural electrodes (Unique Medical, Tokyo, Japan) were implanted subdurally into the participants (Unique Medical, Tokyo, Japan). Each grid or strip comprised a silastic sheet embedded with platinum electrodes of either 1.5 mm or 3.0 mm diameter. The center of each electrode was aligned at 5 mm or 10 mm intervals within the sheet. The location and type of implanted electrodes were determined according to clinical necessity, depending on the suspected epileptic foci. Two participants had electrodes covering the left hemisphere, whereas three participants had electrodes on the right side. The electrodes covered the temporal tip, parahippocampal gyrus, mediobasal and posterobasal parts of the temporal base, and the lateral cortex of the temporal lobe in all participants. Three participants (participants 1, 2, and 3) had depth electrodes implanted in the hippocampus (the interval of the recording site was 5 mm). A head CT scan was performed the day after the electrode implantation surgery. To confirm the locations of the implanted electrodes, CT images were fused with preoperative T1-weighted MRI images to create a 3D model of the brain using volume rendering (Figure 1). This method was described in detail in our previous study (Nagata et al., 2022).

### 2.3 Quantitating GOR from the long-term video ECoG data

The increased regularity of gamma oscillations during the interictal periods can be quantified as a decreased MSE of the gamma frequency band. In this study, MSE was calculated from the interictal resting-state ECoG waveforms as follows: Long-term video ECoG recordings started the day after electrode implantation or later. The ECoG signals were digitally recorded in an electrically shielded room at a sampling rate of 2000 Hz using a 256-channels digital electroencephalogram (EEG-1200 Neurofax, Nihon Koden, Japan). Notably, we reduced the dose of anti-seizure medications during the study period. The reduced dose depended on the participant’s clinical course and seizure frequency (Table 1). From the recorded ECoG data, we extracted 60 min of the interictal period during non-rapid eye movement (NREM) sleep to highlight gamma oscillations, which were expected to be enhanced in correlation with interictal spikes during NREM sleep (Huberfeld et al., 2011; Moore et al., 2021). Moreover, 20 sets of ECoG segments of 20-s blocks were extracted pseudo-randomly for further analysis. We further calculated the entropy of each block using the following methods, and obtained the final results by averaging the 20 blocks. To exclude the impact of epileptic seizures, the analyzed period was set as seizure-free for at least 24 h.

The MSE enables cross-frequency analysis by combining the sample entropies for multiple frequency bands (Richman and Moorman, 2000). The sample entropy of each frequency band represents the complexity of the time series data at a specific frequency. To decompose the recorded ECoG data into a specific frequency and calculate the sample entropy, we introduced a constant defined as *tau*. The recorded ECoG was first down-sampled to 200 Hz, and then coarse-grained by the width of *tau*(*tau* ranges from 1 to 20). From the coarse-grained waveform for each *tau* value, the sample entropy corresponding to 200/*tau* Hz band was calculated according to the following procedure. For example, the sample entropy of a frequency band around 66.7 Hz could be calculated via a *tau* value of three. To calculate the sample entropy, a continuous vector **M**, with a length of *m* time points was derived from a coarse-grained waveform. For all probable pairs of ***M_i_*** and ***M_j_*** (*i j*), Chebyshev distances of the two vectors were calculated. The number of pairs whose Chebyshev distances were below a pre-determined threshold *r* was counted and divided by the number of all probable pairs of vectors **M**, which was defined as *C*_*m*_(*r*).


$$C_{m}\,\left(r\right)=\frac{N_{m}}{\left(N-m+1\right)\,\left(N-m\right)}$$

$$N_{m}=numberofpairsofiandjwith|{\text{M}}_{i}-{\text{M}}_{j}|<r\left(i\neq j\right)$$

*C*_*m* + 1_(*r*) can also be calculated using the vector length *m+1*. The sample entropy corresponding to a constant *tau* (*SE*_*tau*_(*m*,*r*)) was calculated as follows:


$$SE_{tau}\;\left(m,r\right)\;=\text{ log}\left(C_{m\text{+1}}\left(r\right)/C_{m}\left(r\right)\right)$$

Based on previous research concerning the MSE of ECoG in epileptic patients, we took two as the vector length *m*, and 0.2 as the threshold *r* (Sato et al., 2017).

Using the procedure described above, sample entropy values corresponding to the frequency band from 10 Hz (*tau* = 20) to 200 Hz (*tau* = 1) were derived in a stepwise manner. To estimate the MSE of gamma oscillations (30–70 Hz), we averaged the sample entropy corresponding to *tau*, from three (66.7 Hz) to seven (28.6 Hz) (Costa et al., 2002). A decrease in the MSE corresponds to an increase in the GOR, indicating enhanced synchronization of inhibitory interneurons.

### 2.4 CCEP recordings and analysis

CCEP recordings were performed for all participants in addition to routine video ECoG recordings. CCEP recordings were conducted after completing video ECoG recordings for diagnostic purposes at least 24 hours after the last epileptic seizure. For recording, the participants were seated on a reclining bed and instructed to remain awake and open their eyes. The participants continued to take their usual anti-seizure medications.

Adjacent pairs of implanted electrodes were used to apply electrical stimulation to the brain surfaces of the participants. Single-pulse stimulations consisted of a square wave of altering polarity with an amplitude of 8 mA and at a duration of 300 microsecond were delivered at a frequency of 1 Hz for a total of 30 times. The evoked waveforms were recorded for all electrodes except for the two used for stimulation. The CCEP was obtained by averaging 30 responses. We performed this operation for all electrodes by changing the stimulating pair and obtained evoked waveforms for each stimulation site. No clinical or subclinical seizures were observed during the experiment.

The derivation of CCEPs and the method used to evaluate their potential were as follows. The waveform obtained by a single electrical stimulation was cut from the start of the stimulation to 1000 ms after the stimulation (one epoch). Each stimulation pair contained 30 epochs of the waveform. Epochs with excessive noise were excluded from further analyses. For each epoch, the baseline period was set at 655–950 ms and the average was subtracted from the raw waveform. Instead of the pre-stimulus periods, we utilized the post-stimulus data as the baseline because the stimulus charge caused varying degrees of background potential shifts in each trial. CCEP waveforms were obtained by averaging the total number of epochs. The magnitude of the recorded potential in the target period was derived as the root mean square (RMS) of the waveform of the obtained CCEP waveform. The target period for analyzing the RMS was set from 5 ms to 300 ms from the start of the stimulation to include N1 and N2 and remove the impact of the stimulation current itself. This method follows the methodology of a previous study conducted in our laboratory (Shimada et al., 2017).

Shimada et al. showed that a significant portion of the responses measured by electrodes near the stimulation site was caused by the volume-conducted potential (VCP) of the large potential generated directly under the stimulation site (Shimada et al., 2017). In ECoG, the electrical conductivity of the volume conductor is considered constant. In this condition, the fundamental solution of Poisson’s equation and the current dipole model shows that the potential measured at a distant site from the current source decays in proportion to the inverse square of the distance (Zaveri et al., 2009). In this study, we assumed the center coordinates of the electrode pair used for stimulation as the stimulation center and the signal source, and analyzed the waveforms obtained from electrodes located within a radius of 20 mm from the signal source.

The total of N electrodes that satisfied the conditions presented above, φ_*k*_ represented the target RMS measured from an electrode E_*k*_ located at a distance of *r_k_* from the signal source (k represents any natural number from 1 to N). As the potential of the VCP decays with the inverse square of the distance, the potential φ_*k*_ can be converted to φ_*Rk*_ at a virtual electrode defined as E_*R*_ located at a distance *R*from the signal source.


φR⁢k=φk×rk2R2

To approximate the VCP recorded at the electrode E_*R*_, we calculated φ_*Rk*_ for each electrode, and determined the average as follows:


∅R=1N⁢∑k=1Nφk×rk2R2

This facilitated a quantitative comparison of the magnitude of the VCP generated by stimulation at different cortical locations. In the present study, we set *R* to 10 mm from the stimulation center and used ∅_*R*_ as a measure of CCEP in the vicinity of the stimulation site. In this study, ∅_*R*_ equates to the CCEP reactivity.

## 3 Results

### 3.1 Gamma oscillation regularity measured by multiscale entropy

**FIGURE 2:**
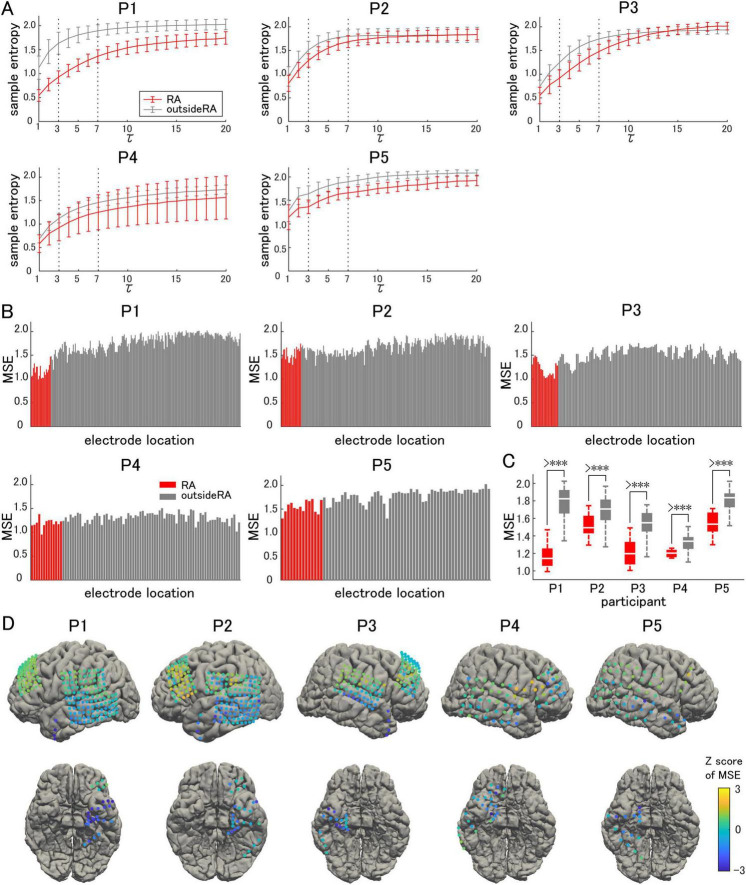
Sample entropy and multiscale entropy. **(A)** The sample entropy corresponding to each frequency band is shown on the vertical axis, with 200/*tau* Hz corresponding to a constant *tau*. Measurements in the resection area (RA: red lines) are compared with those outside of the resection area (gray lines). **(B)** Multiscale entropy (MSE) is compared on an electrode-by-electrode basis. **(C)** The averaged MSE in the RA is significantly lower than that outside the RA. **(D)** The Z-score of the MSE in each electrode is visualized on individual brains as color maps. The symbol *** indicates *p*-values of less than 0.05.

The sample entropy of each frequency band was calculated for all recorded electrodes, and averaged separately for the resection area and area outside the resection (Figure 2A). The resection area was defined as the region where the surgical procedures were performed. The gamma band was defined as *tau* ranging from 3 to 7, corresponding to frequencies between 66.7 Hz and 28.6 Hz. For all the participants, the sample entropy increased in a curved manner as the frequency decreased. The largest difference was observed in the gamma band.

The average sample entropy for the gamma band, *tau* ranging from 3 to 7, was used to calculate the MSE for each electrode. For each participant, the MSE for each electrode location was compared, charted, and illustrated as a color map on a 3D model of the brain surface (Figures 2B, D). A decrease in MSE was observed from the temporal pole to the hippocampus and parahippocampal gyrus, while it tended to be maintained outside the resection area. The average MSE in the resection area was significantly lower in all participants (paired t-test, two-tailed, Figure 2C), indicating an increase in the GOR and enhanced synchronization of inhibitory interneuron activities. This tendency varied among the participants, with a particularly significant decrease observed in Participant 1.

### 3.2 CCEP reactivity

**FIGURE 3:**
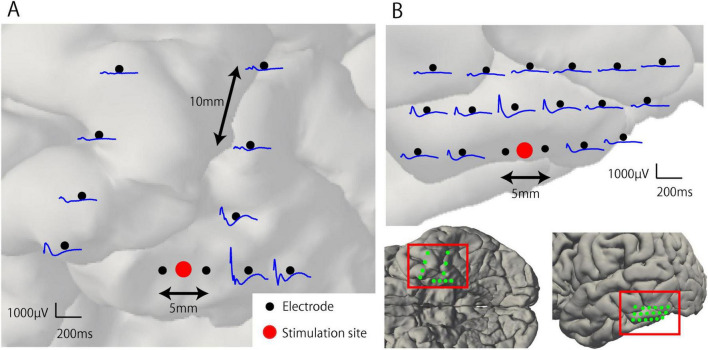
Representative waveforms of cortico-cortical evoked potential near the stimulation site (Participant 2). **(A)** Electrical stimulation was applied to the anterior pair of the mesial temporal lobe electrodes within the resection area (RA). Early and large components of the CCEP waveforms were observed around the stimulation site and attenuated with distance. **(B)** Electrical stimulation was applied to the inferior temporal gyrus located outside the RA. The CCEP response observed around the stimulation site is smaller than that within the RA.

**FIGURE 4:**
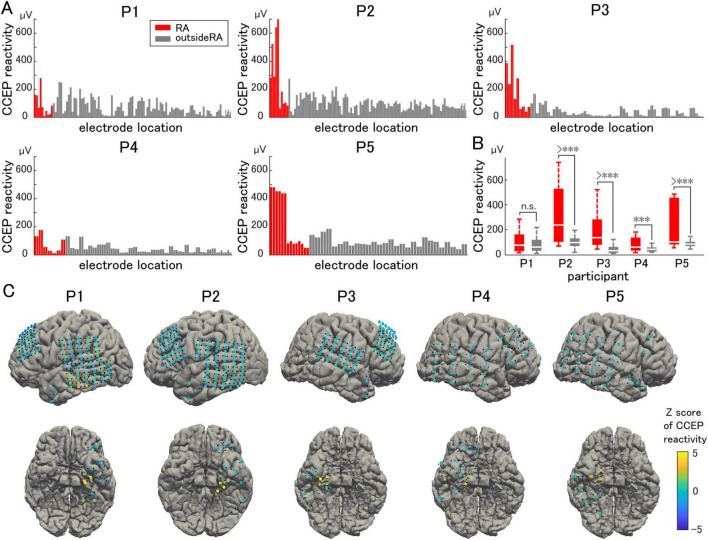
Cortico-cortical evoked potential reactivity. **(A)** Cortico-cortical evoked potential (CCEP) reactivity in the vicinity of the stimulation site is compared electrode by electrode. **(B)** The averaged CCEP reactivity in the resection area (RA) is significantly higher than that outside of the RA (except for Participant 1). **(C)** The Z-score of the CCEP reactivity of each electrode is visualized on individual brains as color maps. The symbol *** indicates *p*-values of less than 0.05.

Representative waveforms of CCEP recorded near the stimulation site (Participant 2) are shown in Figure 3. The CCEP reactivity was calculated as the RMS during the target period and illustrated using a color map of the brain surface (Figures 4A, C). CCEP reactivity values obtained by electrical stimulation were assigned to each stimulation electrode pair. Participants 2, 3, and 5 showed conspicuously high CCEP reactivity values in the hippocampus and parahippocampal gyrus, suggesting increased excitability, whereas CCEP reactivity outside the resection area tended to be low. With the exception of Participant 1, statistically significant differences in CCEP reactivity were observed within the resection area compared to outside the resection area (paired t-test, two-tailed, Figure 4B).

### 3.2 Excitatory and Inhibitory (EI) index

**FIGURE 5:**
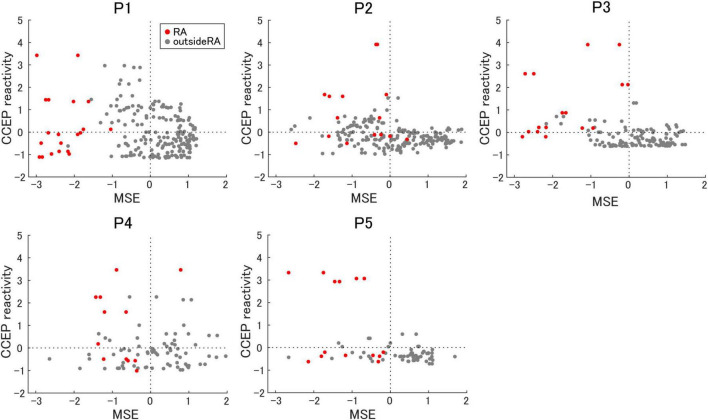
The relationship between multiscale entropy and cortico-cortical evoked potential reactivity. The correlation between multiscale entropy (MSE) and cortico-cortical evoked potential (CCEP) reactivity is shown. Electrodes in the resection area (RA) are represented as red dots.

To evaluate the relationship among epileptogenicity, MSE, and CCEP reactivity, the results were standardized and converted to within-participant Z-scores to align their scales. Figure 5 presents a scatter plot of the MSE on the horizontal axis and the CCEP reactivity on the vertical axis. According to this hypothesis, a lower MSE indicates increased inhibition, whereas a higher CCEP reactivity indicates increased excitation, both of which are indicative of epileptogenicity.

Using these Z-scoring parameters, we defined the EI index using the following formula:


$$E\,I\,i\,n\,d\,e\,x=Z_{C\,C\,E\,P}-Z_{M\,S\,E}$$

**FIGURE 6:**
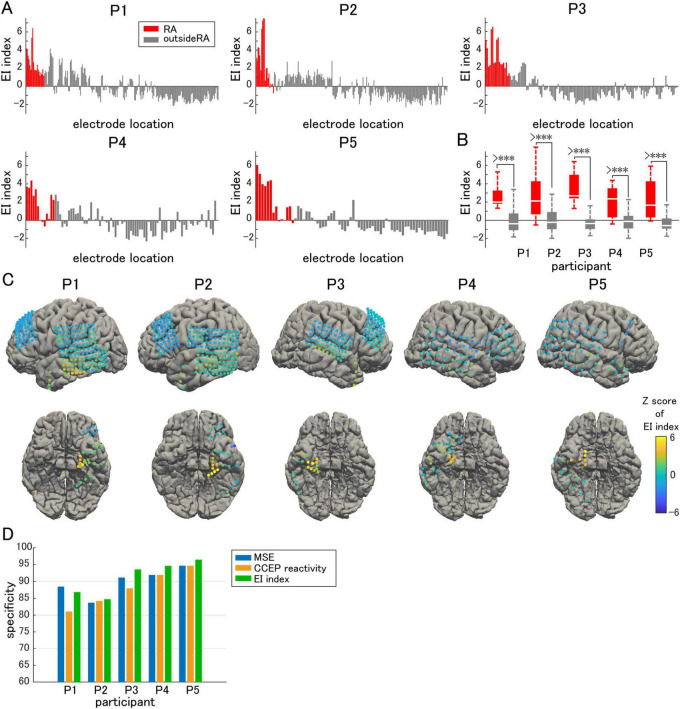
EI index. **(A)** EI index is compared electrode by electrode. **(B)** The averaged EI index in the resection area (RA) is significantly higher than that outside of the RA. **(C)** The Z-score of the EI index in each electrode is visualized on individual brains as color maps. **(D)** The EI index predicted the epileptogenic zone with higher specificity than either multiscale entropy (MSE) or cortico-cortical evoked potential (CCEP) reactivity alone. The symbol *** indicates *p*-values of less than 0.05.

By combining these two parameters, we intended to evaluate the effects of both the inhibitory and excitatory activities to comprehensively evaluate epileptogenicity (Figures 6A, C). A significantly higher EI index was observed in the resection area than outside the resection area in all participants (Figure 6B). Next, we sorted the EI index in descending order, and defined the cutoff value as the rank above the number of electrodes in the resection area. Above the cutoff rank, the number of electrodes outside the resection area was counted to calculate the false-positive rate. The specificity of the EI index in predicting the actual resection area was demonstrated. The same procedure was performed for both MSE and CCEP reactivity by sorting the MSE in ascending order and the CCEP reactivity in descending order. Since only seizure-free participants following focal resection surgery were included in this study, we assumed that the epileptogenic zone was contained within the resection area. Thus, the specificity for predicting the resection area and that for predicting the epileptogenic zone can be treated equally. Although a relatively high specificity for predicting the epileptogenic zone was achieved using either MSE or CCEP reactivity, in all subjects except Participant 1, EI index was slightly more specific in predicting the resection area than either MSE or CCEP reactivity alone (Figure 6D).

## 4 Discussion

### 4.1 Summary

In this study, we aimed to spatially evaluate excitatory and inhibitory activities using two quantitative parameters. A decrease in MSE, indicating an elevation in the GOR, was observed around the EZ. As reported in previous studies, this response corresponds to the enhancement of inhibitory interneuronal activity mediated by GABA. However, in contrast, an increase in the CCEP reactivity near the stimulation site was observed in the narrower region surrounding the SOZ. Previous studies have indicated that this response may indicate excitatory cortical activity predominantly mediated by cortical pyramidal cell activation. Although these parameters could be used independently to predict epileptogenic regions with relatively high specificity, combining them further improved predictive accuracy.

### 4.2 Significance

Excitability refers to the intrinsic capability of neurons to generate action potentials in response to a given stimulus, a concept that includes both excitatory and inhibitory neuronal activity. Single-pulse electrical stimulation and transcranial magnetic stimulation have been applied to evaluate cortical excitability (Abbruzzese and Trompetto, 2002). Excitatory activity, on the other hand, refers to depolarizing activity of postsynaptic neurons mediated by excitatory neurotransmitters. Excitability and excitatory activity are, therefore, distinct concepts. For instance, it is known that neurons shift to hyperpolarization and overall excitatory activity is reduced during non-rapid eye movement sleep, while excitability to stimuli is enhanced (Usami et al., 2015). CCEP serves as an indicator of excitability by incorporating both excitatory and inhibitory activities, though these activities exhibit distinct temporal patterns. Kobayashi et al. demonstrated that high gamma activity associated with CCEP is elevated at the N1 peak and diminished at the N2 peak compared to the baseline (Kobayashi et al., 2017). This response reflects brief synchronous burst of pyramidal cells followed by prolonged suppression, suggesting a mechanism analogous to that observed in interictal discharges (Alarcón et al., 2012). Interictal epileptic discharges generates epileptic synchrony through intrinsic or synaptically driven synchronous excitatory activity of pyramidal cells (Hofer et al., 2022). It is also proposed that excitatory neuronal activity observed in the early stage of CCEP is akin to the intrinsic cortical activity of feed-forward input (Kunieda et al., 2015). These homologies suggests that the early response of the CCEP may also serve as an indicator of excitatory activity in the epileptic brain.

In this study, the difference in the spatial distribution of the two parameters was characteristic: enhanced CCEP reactivity was localized to a restricted region centered on the seizure onset region, whereas GOR elevation was observed in a broader region surrounding the SOZ. Previous studies on the GOR have postulated that during the interictal period, the brains of patients with epilepsy exhibit increased synchronization of inhibitory interneurons in the seizure-onset region. Conversely, desynchronization of interneurons is relatively preserved in the EZ surrounding the SOZ, with a moderate increase in the GOR during the interictal period (Sato et al., 2022; Sato et al., 2017). Their study proposed a model in which seizures were suppressed in the SOZ by inhibitory interneurons, mitigating the marked excitatory activity. They further hypothesized that excitatory activity exceeds inhibitory one when this fragile balance is disrupted, leading to preictal and ictal states. One aim of the present study was to derive this excitatory activity from an electrophysiological modality other than the GOR. In the present study, the CCEP reactivity was enhanced in a narrower region near the SOZ than in the region of GOR enhancement, which is consistent with the characteristics of excitatory activity in the aforementioned hypothesis. As discussed in the Introduction, although excitability is a concept that includes both excitatory and inhibitory activities, we assumed that excitatory activity was the primary component of CCEP reactivity near the stimulation site. The results of the present study supported our hypothesis, indicating the utility of CCEP reactivity as an indicator of local cortical excitatory activity in MTLE patients.

The results of this study also suggest that the EZ may be identified from ECoG findings during the interictal period. The EZ encompasses the area in which resection results in complete seizure cessation (Lüders et al., 2006). This concept is retrospectively defined based on seizure prognosis following focal resection, and there is currently no established method to accurately identify EZs based solely on preoperative evaluation. The gold standard for EZ localization is capturing ictal EEG or ECoGs through long-term recordings. However, this approach is burdensome for both clinicians and patients. Thus, a different method capable of accurately identifying the EZ based on interictal findings is needed.

High-frequency oscillations (HFOs) have previously been proposed as an indicator of cortical excitability and are expected to act as a biomarker for EZ (Zijlmans et al., 2009), but HFO-guided resection has not been shown to improve seizure outcomes (Gloss et al., 2017; Jacobs et al., 2018; Zweiphenning et al., 2022). Various modalities have been proposed as potential biomarkers for detecting EZs; however, no single method has been established to replace the gold standard.

The present study was designed based on the idea that combining multiple indicators related to cortical excitatory and inhibitory activity could provide greater accuracy in localizing EZs. Our results revealed inter-subject quantitative variations in GOR and CCEP reactivity within the resection area. For example, Participant 1 showed a significant increase in GOR but no significant change in CCEP reactivity; Participant 3 showed significant changes in both parameters; and Participant 4 demonstrated only mild changes in both parameters, but a clear increase in the EI index within the resection area. These findings indicate that the balance between excitatory and inhibitory activities varies, even within the same type of MTLE. Combining multiple indicators to evaluate heterogeneous pathologies as an imbalance in the EI interplay may assist in predicting the EZ with greater accuracy in MTLE.

### 4.3 Limitations

This study has several limitations. First, the variations in the responsiveness of each cortical structure to electrical stimulation should be considered. The CCEP reactivity of the SOZ was higher than that of the non-SOZ in both temporal and extra-temporal lobe epilepsy. In contrast, CCEP reactivity in the SOZ in patients with extratemporal lobe epilepsy is not higher than that in the temporal lobe cortex of non-SOZ in temporal lobe epilepsy (Iwasaki et al., 2010). This may be attributed to the distinct connectivity profiles between the medial temporal lobe and other cortices. Although we approximated from previous studies that the majority of CCEP reactivity recorded near the stimulation site were VCPs of excitatory activity arising from the stimulated cortex, we cannot exclude the influence of the connectivity profile from local and short-range cortical-subcortical projections. The influence of the local connectivity may vary depending on the anatomical site. This indicates the possibility that CCEP reactivity may be overestimated in the medial temporal lobe.

At this time, the results obtained are applicable only to the MTLE. Further investigations with a broader range of participants with greater variation in epileptic foci are required to determine whether the results of this study could be generalized to patients with extratemporal lobe epilepsy. However, we were compelled to limit our analysis to patients with MTLE because we were unable to secure a sufficient number of study participants with extratemporal lobe epilepsy. The small and biased sample size is the most significant limitation of the present study.

Second, this study focused only on patients with good seizure prognosis (Engel class 1). To estimate the accuracy of the EI index in predicting seizure prognosis in patients with MTLE, patients with a poor prognosis must also be included in future studies. However, the ground truth of the epileptogenic zone can only be clinically determined as “the area included in the resection zone of participants with good prognosis,” making it difficult to accurately define EZ in those with poor seizure prognosis. Anterior temporal lobectomy is a relatively standardized surgical procedure, in which the anterior temporal lobe is typically resected en bloc, even in the absence of epileptogenicity at the tip of the temporal lobe or uncus. Therefore, the true EZ may have been narrower than that defined by the surgical resection area in this study. The use of specificity, rather than sensitivity, as an evaluation metric in this study was due to these circumstances. Further refinement of the study design is necessary to determine whether the GOR and CCEP reactivity could influence the surgical prognosis of MTLE.

Third, the choice of the baseline period of the CCEP should be carefully considered. In the present study, we selected the 655–950 ms post-stimulus period as the baseline to minimize the influence of background potential shifts. However, it is important to note that prolonged responses lasting up to one second after electrical stimulation are also recorded in CCEP (Paulk et al., 2022), as well as delayed responses that have been reported in epilepsy patients, presumably reflecting excitability (Valentín et al., 2002). These responses may have potentially influenced the outcomes of this study via baseline selection.

Finally, it is important to consider that GOR and CCEP are not independent indicators that can directly and exclusively reflect inhibitory and excitatory neuronal activities. These conflicting activities are intrinsically inseparable in electrocorticography, in which the summation of the electrical activity of the neural cell population beneath the recording electrode can be obtained. The interplay between the activities of pyramidal cells and inhibitory interneurons should be confirmed through basic research at the cellular or local network levels.

## 5 Conclusion

In this study, using the ECoG recordings of patients with MTLE, cortical inhibitory activity was quantified using the GOR derived from MSE, whereas excitatory activity was assessed using CCEP responses in the vicinity of the stimulation site. The spatial relationship between the two parameters and the EZ was demonstrated, and the index combining both the GOR and CCEP reactivity exhibited high accuracy in predicting the EZ. Further studies are required to generalize the results to extra-temporal lobe epilepsy and to establish these two parameters as potential biomarkers for determining the EZ from interictal ECoG recordings.

## Data Availability

The original contributions presented in the study are included in the article/supplementary material, further inquiries can be directed to the corresponding author.

## References

[B1] Abbruzzese G. Trompetto C. (2002). Clinical and research methods for evaluating cortical excitability. *J. Clin. Neurophysiol.* 19 307–321. 10.1097/00004691-200208000-00005 12436087

[B2] Alarcón G. Martinez J. Kerai S. V. Lacruz M. E. Quiroga R. Q. Selway R. P. (2012). In vivo neuronal firing patterns during human epileptiform discharges replicated by electrical stimulation. *Clin. Neurophysiol.* 123 1736–1744. 10.1016/j.clinph.2012.02.062 22410162 PMC3432232

[B3] Ando M. Nobukawa S. Kikuchi M. Takahashi T. (2021). Identification of electroencephalogram signals in Alzheimer’s disease by multifractal and multiscale entropy analysis. *Front. Neurosci.* 15:667614. 10.3389/fnins.2021.667614 34262427 PMC8273283

[B4] Bradford H. F. (1995). Glutamate, GABA and epilepsy. *Prog. Neurobiol.* 47 477–511. 10.1016/0301-0082(95)00030-5 8787032

[B5] Buzsáki G. Wang X. J. (2012). Mechanisms of gamma oscillations. *Annu. Rev. Neurosci.* 35 203–225. 10.1146/annurev-neuro-062111-150444 22443509 PMC4049541

[B6] Buzsáki G. Leung L. W. Vanderwolf C. H. (1983). Cellular bases of hippocampal EEG in the behaving rat. *Brain Res.* 287 139–171. 10.1016/0165-0173(83)90037-1 6357356

[B7] Cardin J. A. Carlén M. Meletis K. Knoblich U. Zhang F. Deisseroth K. (2009). Driving fast-spiking cells induces gamma rhythm and controls sensory responses. *Nature* 459 663–667. 10.1038/nature08002 19396156 PMC3655711

[B8] Costa M. Goldberger A. L. Peng C. K. (2002). Multiscale entropy analysis of complex physiologic time series. *Phys. Rev. Lett.* 89:068102. 10.1103/PhysRevLett.89.068102 12190613

[B9] Enatsu R. Piao Z. O’Connor T. Horning K. Mosher J. Burgess R. (2012). Cortical excitability varies upon ictal onset patterns in neocortical epilepsy: A cortico-cortical evoked potential study. *Clin. Neurophysiol.* 123 252–260. 10.1016/j.clinph.2011.06.030 21802356

[B10] Engel J. Jr. (1995). Inhibitory mechanisms of epileptic seizure generation. *Adv. Neurol.* 67 157–171. 8848967

[B11] Engel J. Jr. (1996). Excitation and inhibition in epilepsy. *Can. J. Neurol. Sci.* 23 167–174. 10.1017/s0317167100038464 8862837

[B12] Gloss D. Nevitt S. J. Staba R. (2017). The role of high-frequency oscillations in epilepsy surgery planning. *Cochrane Database Syst. Rev.* 10:Cd010235. 10.1002/14651858.CD010235.pub3 28981957 PMC6485644

[B13] Hofer K. T. Kandrács Á Tóth K. Hajnal B. Bokodi V. Tóth E. Z. (2022). Bursting of excitatory cells is linked to interictal epileptic discharge generation in humans. *Sci. Rep.* 12:6280. 10.1038/s41598-022-10319-4 35428851 PMC9012754

[B14] Huberfeld G. Menendez de la Prida L. Pallud J. Cohen I. Le Van Quyen M. Adam C. (2011). Glutamatergic pre-ictal discharges emerge at the transition to seizure in human epilepsy. *Nat. Neurosci.* 14 627–634. 10.1038/nn.2790 21460834

[B15] Iwasaki M. Enatsu R. Matsumoto R. Novak E. Thankappen B. Piao Z. (2010). Accentuated cortico-cortical evoked potentials in neocortical epilepsy in areas of ictal onset. *Epileptic Disord.* 12 292–302. 10.1684/epd.2010.0334 20952353

[B16] Jacobs J. Wu J. Y. Perucca P. Zelmann R. Mader M. Dubeau F. (2018). Removing high-frequency oscillations: A prospective multicenter study on seizure outcome. *Neurology* 91 e1040–e1052. 10.1212/wnl.0000000000006158 30120133 PMC6140372

[B17] Kobayashi K. Matsumoto R. Matsuhashi M. Usami K. Shimotake A. Kunieda T. (2017). High frequency activity overriding cortico-cortical evoked potentials reflects altered excitability in the human epileptic focus. *Clin. Neurophysiol.* 128 1673–1681. 10.1016/j.clinph.2017.06.249 28750290

[B18] Köhling R. Vreugdenhil M. Bracci E. Jefferys J. G. (2000). Ictal epileptiform activity is facilitated by hippocampal GABAA receptor-mediated oscillations. *J. Neurosci.* 20 6820–6829. 10.1523/jneurosci.20-18-06820.2000 10995826 PMC6772821

[B19] Kunieda T. Yamao Y. Kikuchi T. Matsumoto R. (2015). New approach for exploring cerebral functional connectivity: Review of cortico-cortical evoked potential. *Neurol. Med. Chir.* 55 374–382. 10.2176/nmc.ra.2014-0388 25925755 PMC4628165

[B20] Lüders H. O. Najm I. Nair D. Widdess-Walsh P. Bingman W. (2006). The epileptogenic zone: General principles. *Epileptic Disord.* 8 S1–S9. 17012067

[B21] Mann E. O. Paulsen O. (2007). Role of GABAergic inhibition in hippocampal network oscillations. *Trends Neurosci.* 30 343–349. 10.1016/j.tins.2007.05.003 17532059

[B22] Matsumoto J. Y. Stead M. Kucewicz M. T. Matsumoto A. J. Peters P. A. Brinkmann B. H. (2013). Network oscillations modulate interictal epileptiform spike rate during human memory. *Brain* 136 2444–2456. 10.1093/brain/awt159 23803305 PMC3722348

[B23] Matsumoto R. Kinoshita M. Taki J. Hitomi T. Mikuni N. Shibasaki H. (2005). In vivo epileptogenicity of focal cortical dysplasia: A direct cortical paired stimulation study. *Epilepsia* 46 1744–1749. 10.1111/j.1528-1167.2005.00284.x 16302853

[B24] Matsumoto R. Nair D. R. LaPresto E. Najm I. Bingaman W. Shibasaki H. (2004). Functional connectivity in the human language system: A cortico-cortical evoked potential study. *Brain* 127 2316–2330. 10.1093/brain/awh246 15269116

[B25] Moore J. L. Carvalho D. Z. St Louis E. K. Bazil C. (2021). Sleep and Epilepsy: A focused review of pathophysiology, clinical syndromes, co-morbidities, and therapy. *Neurotherapeutics* 18 170–180. 10.1007/s13311-021-01021-w 33786803 PMC8116418

[B26] Nagata K. Kunii N. Shimada S. Fujitani S. Takasago M. Saito N. (2022). Spatiotemporal target selection for intracranial neural decoding of abstract and concrete semantics. *Cereb. Cortex* 32 5544–5554. 10.1093/cercor/bhac034 35169837 PMC9753048

[B27] Paulk A. C. Zelmann R. Crocker B. Widge A. S. Dougherty D. D. Eskandar E. N. (2022). Local and distant cortical responses to single pulse intracranial stimulation in the human brain are differentially modulated by specific stimulation parameters. *Brain Stimul.* 15 491–508. 10.1016/j.brs.2022.02.017 35247646 PMC8985164

[B28] Ren L. Kucewicz M. T. Cimbalnik J. Matsumoto J. Y. Brinkmann B. H. Hu W. (2015). Gamma oscillations precede interictal epileptiform spikes in the seizure onset zone. *Neurology* 84 602–608. 10.1212/wnl.0000000000001234 25589669 PMC4335986

[B29] Richman J. S. Moorman J. R. (2000). Physiological time-series analysis using approximate entropy and sample entropy. *Am. J. Physiol. Heart Circ. Physiol.* 278 H2039–H2049. 10.1152/ajpheart.2000.278.6.H2039 10843903

[B30] Sato Y. Tsuji Y. Kawauchi Y. Iizuka K. Kobayashi Y. Irie R. (2021). Epileptogenic zone localization using intraoperative gamma oscillation regularity analysis in epilepsy surgery for cavernomas: Patient series. *J. Neurosurg. Case Lessons* 1:Case20121. 10.3171/case20121 36033917 PMC9394110

[B31] Sato Y. Tsuji Y. Yamazaki M. Fujii Y. Shirasawa A. Harada K. (2022). Interictal high gamma oscillation regularity as a marker for presurgical epileptogenic zone localization. *Oper. Neurosurg.* 23 164–173. 10.1227/ons.0000000000000245 35486873

[B32] Sato Y. Wong S. M. Iimura Y. Ochi A. Doesburg S. M. Otsubo H. (2017). Spatiotemporal changes in regularity of gamma oscillations contribute to focal ictogenesis. *Sci. Rep.* 7:9362. 10.1038/s41598-017-09931-6 28839247 PMC5570997

[B33] Scharfman H. E. (2007). The neurobiology of epilepsy. *Curr. Neurol. Neurosci. Rep.* 7 348–354. 10.1007/s11910-007-0053-z 17618543 PMC2492886

[B34] Shimada S. Kunii N. Kawai K. Matsuo T. Ishishita Y. Ibayashi K. (2017). Impact of volume-conducted potential in interpretation of cortico-cortical evoked potential: Detailed analysis of high-resolution electrocorticography using two mathematical approaches. *Clin. Neurophysiol.* 128 549–557. 10.1016/j.clinph.2017.01.012 28226289

[B35] Takahashi T. Cho R. Y. Murata T. Mizuno T. Kikuchi M. Mizukami K. (2009). Age-related variation in EEG complexity to photic stimulation: A multiscale entropy analysis. *Clin. Neurophysiol.* 120 476–483. 10.1016/j.clinph.2008.12.043 19231279 PMC2880484

[B36] Treiman D. M. (2001). GABAergic mechanisms in epilepsy. *Epilepsia* 42 8–12. 10.1046/j.1528-1157.2001.042suppl.3008.x 11520315

[B37] Tukker J. J. Fuentealba P. Hartwich K. Somogyi P. Klausberger T. (2007). Cell type-specific tuning of hippocampal interneuron firing during gamma oscillations in vivo. *J. Neurosci.* 27 8184–8189. 10.1523/jneurosci.1685-07.2007 17670965 PMC6673067

[B38] Usami K. Matsumoto R. Kobayashi K. Hitomi T. Shimotake A. Kikuchi T. (2015). Sleep modulates cortical connectivity and excitability in humans: Direct evidence from neural activity induced by single-pulse electrical stimulation. *Hum. Brain Mapp.* 36 4714–4729. 10.1002/hbm.22948 26309062 PMC6869089

[B39] Valentín A. Anderson M. Alarcón G. Seoane J. J. Selway R. Binnie C. D. (2002). Responses to single pulse electrical stimulation identify epileptogenesis in the human brain in vivo. *Brain* 125 1709–1718. 10.1093/brain/awf187 12135963

[B40] Zaveri H. P. Duckrow R. B. Spencer S. S. (2009). Concerning the observation of an electrical potential at a distance from an intracranial electrode contact. *Clin. Neurophysiol.* 120 1873–1875. 10.1016/j.clinph.2009.08.001 19748311

[B41] Ziburkus J. Cressman J. R. Barreto E. Schiff S. J. (2006). Interneuron and pyramidal cell interplay during in vitro seizure-like events. *J. Neurophysiol.* 95 3948–3954. 10.1152/jn.01378.2005 16554499 PMC1469233

[B42] Zijlmans M. Jacobs J. Zelmann R. Dubeau F. Gotman J. (2009). High-frequency oscillations mirror disease activity in patients with epilepsy. *Neurology* 72 979–986. 10.1212/01.wnl.0000344402.20334.81 19289737 PMC3797085

[B43] Zweiphenning W. Klooster M. A. V. van Klink N. E. C. Leijten F. S. S. Ferrier C. H. Gebbink T. (2022). Intraoperative electrocorticography using high-frequency oscillations or spikes to tailor epilepsy surgery in the Netherlands (the HFO trial): A randomised, single-blind, adaptive non-inferiority trial. *Lancet Neurol.* 21 982–993. 10.1016/s1474-4422(22)00311-8 36270309 PMC9579052

